# Roll-Out Implementation Optimization (ROIO) trial to develop and test the implementation of a food security screening and referral process: a study protocol

**DOI:** 10.1186/s43058-026-01025-7

**Published:** 2026-06-19

**Authors:** Kyung E. Rhee, Sarah Hiller-Venegas, Clare Viglione, Michelle A. Polich, Jeannie S. Huang, Jane J. Kim, Mandeep S. Bajwa, Michelle Zive, Dawn M. Eichen, David R. Strong, Nicole A. Stadnick, Lauren Brookman-Frazee

**Affiliations:** 1 Department of Pediatrics, University of California San Diego, La Jolla, CA USA; 2 Rady Children’s Health, San Diego, CA USA; 3 Herbert Wertheim School of Public Health, University of California San Diego, La Jolla, CA USA; 4 Department of Psychiatry, University of California San Diego, La Jolla, CA USA; 5 UC San Diego ACTRI Dissemination and Implementation Science Center, La Jolla, CA USA; 6 Child and Adolescent Services Research Center, San Diego, CA USA; 7 Department of Pediatrics, UC San Diego School of Medicine, 9500 Gilman Drive, MC 0874, La Jolla, CA 92093 USA

**Keywords:** Food insecurity, Nutrition insecurity, Pediatrics, Roll-Out Implementation and Optimization (ROIO) trial, PRISM, RE-AIM

## Abstract

**Background:**

Food insecurity (FI) is associated with poorer physical and mental health outcomes, exacerbation of chronic diseases, and decreased access to healthcare. Children experiencing FI face additional risks, including lower psychosocial functioning and reduced academic achievement. Although national initiatives call for integrating nutrition support services into healthcare, clinical workflows for FI screening and referral remain inconsistently implemented and difficult to sustain. The goal of this study is to refine, adapt, and optimize a comprehensive FI screening and referral program, and identify effective implementation strategies for pediatric healthcare systems.

**Methods:**

We will use a RollOut Implementation and Optimization (ROIO) trial design to iteratively implement and refine the implementation strategies for our FI screening and referral program (I-FRESH: Implementing Food Referrals for Equity and Sustained Health) across 4 pediatric clinics. I-FRESH includes: (1) FI screening; (2) assessment of family needs and readiness to access services; (3) referral and navigation support to community nutrition programs; and (4) follow-up to assess fit, utilization, and ongoing needs. The Pragmatic, Robust Implementation and Sustainability Model (PRISM) will guide adaptation and evaluation of contextual determinants, while RE-AIM will guide assessment of outcomes (implementation feasibility and acceptability, fidelity, adoption, reach, effectiveness, and maintenance). We will identify several implementation strategies to increase the likelihood that I-FRESH can be successfully implemented and sustained in a pediatric healthcare system. We will enroll 240 participants and assess preliminary effectiveness on family‑level food security and pediatric nutrition‑related health outcomes (e.g. weight status, blood pressure, lipids, HbA1c, liver function tests). The information gathered in this trial will be utilized in the development of a fully powered Type 2 Hybrid Effectiveness-Implementation Trial that will test the effectiveness of the identified implementation strategies and impact of the FI program on clinical outcomes.

**Discussion:**

By integrating PRISM and RE-AIM within an iterative ROIO design, this study will generate a scalable, contextresponsive implementation model for addressing FI in pediatric healthcare settings. Findings will inform sustainable strategies that link families to highquality nutrition support programs and improve nutritionrelated health outcomes for lowincome children.

**Trial registration:**

NCT06661538.

Contributions to the literature • The Roll-Out Implementation and Optimization trial design allows us to quickly and continuously learn and adapt workflows across sites and identify effective strategies to implement a food insecurity screening and referral process that is not confounded by time and system-level changes.

• This design allows us to examine the impact of strategies within clinics over time and between clinics at each time point.

• This study uses community engagement methods to gather healthcare providers and staff, community-based nutrition support programs, and implementation science researchers to address the complex issue of connecting patients with health-related social-needs to appropriate community programs.

## Background

Food insecurity (FI), defined as limited or uncertain access to adequate food [1], is associated with poorer physical and mental health outcomes in adults [2–4], greater financial hardship, and decreased access to care [5]. Children experiencing FI also have poorer health outcomes [6], lower psychosocial functioning [7–9], and lower academic achievement [10]. FI exacerbates chronic health conditions such as liver disease and cardiovascular disease (CVD) [2, 11]. Furthermore, individuals with obesity and poor dietary intake have greater likelihood of developing type 2 diabetes mellitus (T2D), CVD, and cancer [12]. Given the wide reaching impact of good nutrition, Healthy People 2030 identified reducing FI as a top priority to improve health [13].

Access to healthy foods, such as fresh fruits and vegetables, whole grains, nuts, and lean proteins (e.g., Mediterranean diet, DASH diet) are associated with reduced incidence of CVD, T2D, cancer, neurodegenerative disease, and overall mortality [14–16]. Food assistance programs, such as Supplemental Nutrition Assistance Program (SNAP), may moderate these health risks by increasing access to food for those with limited incomes [17]. Other programs, including WIC, school meal programs, and community-based food assistance programs such as food banks also improve access to food, dietary quality, and school-related and developmental outcomes for children [18–21].

In recent years, healthcare systems have increasingly been called upon to implement screening for a wide range of adverse social determinants of health (SDOH). In 2022, The White House Conference on Hunger, Nutrition, and Health underscored the critical role of integrating nutrition support programs and education into the healthcare setting to address food security [22]. To achieve this integration, healthcare systems are encouraged to follow a two-step process: 1) identify the unmet social need and 2) link patients to appropriate services [23]. Despite the simplicity and clarity of this charge, this workflow can be challenging to implement with little consensus on the most effective and sustainable ways to integrate these procedures into current clinical workflows [24–26].

Several barriers hinder successful screening and referral processes in the clinical setting, including technical challenges, difficulties in maintaining fidelity within complex workflows, and constraints on personnel. Additionally, connecting families with community-based programs can be challenging, particularly when there is limited knowledge among healthcare providers about which programs are reliable and provide high-quality services [27]. Furthermore, simply providing program information to families may not be effective. Without comprehensive service navigation (e.g., by a social worker or care navigator who actively assists families in accessing programs) or follow-up communication from community organizations to confirm program utilization, it remains unclear whether referrals alone lead to meaningful health improvements [24, 27].

**Fig. 1:**
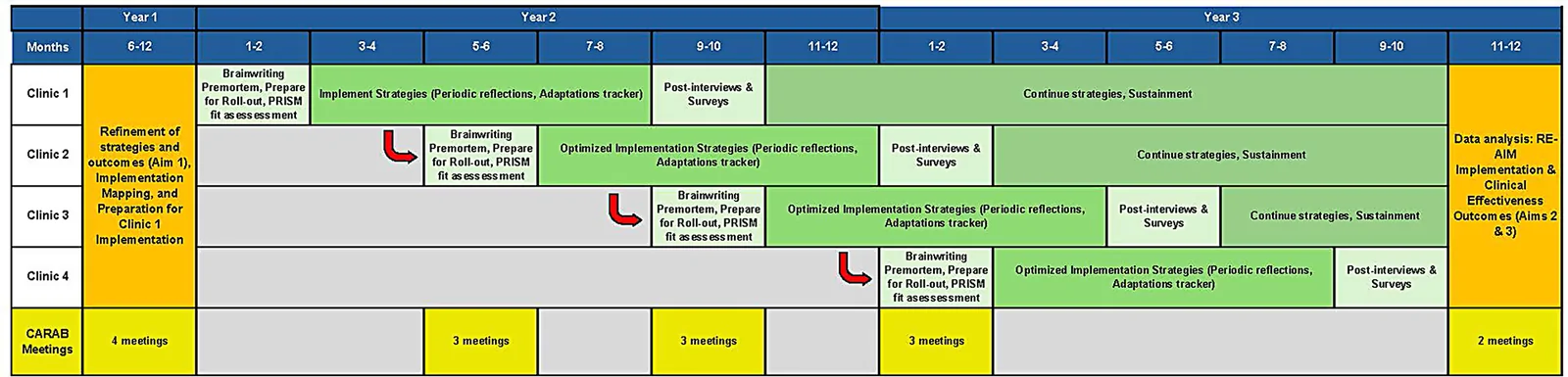
Roll-Out Implementation and Optimization design for the I-FRESH trial. Four clinics will participate in this roll-out implementation and optimization trial. Refinement and adaptation of strategies will occur before implementing I-FRESH in each clinic, and will be based on learnings from the prior clinic’s implementation effort. Implementation and clinical effectiveness outcomes will be measured across each clinic. The Pragmatic, Robust Implementation and Sustainability Model (PRISM) will be used to guide the refinement, optimization, implementation, and evaluation process, and the Reach, Effectiveness, Adoption, Implementation, and Maintenance (RE-AIM) outcomes framework will be used to evaluate core implementation outcomes, including feasibility, acceptability, fidelity, adoption, reach, and effectiveness

**Fig. 2:**
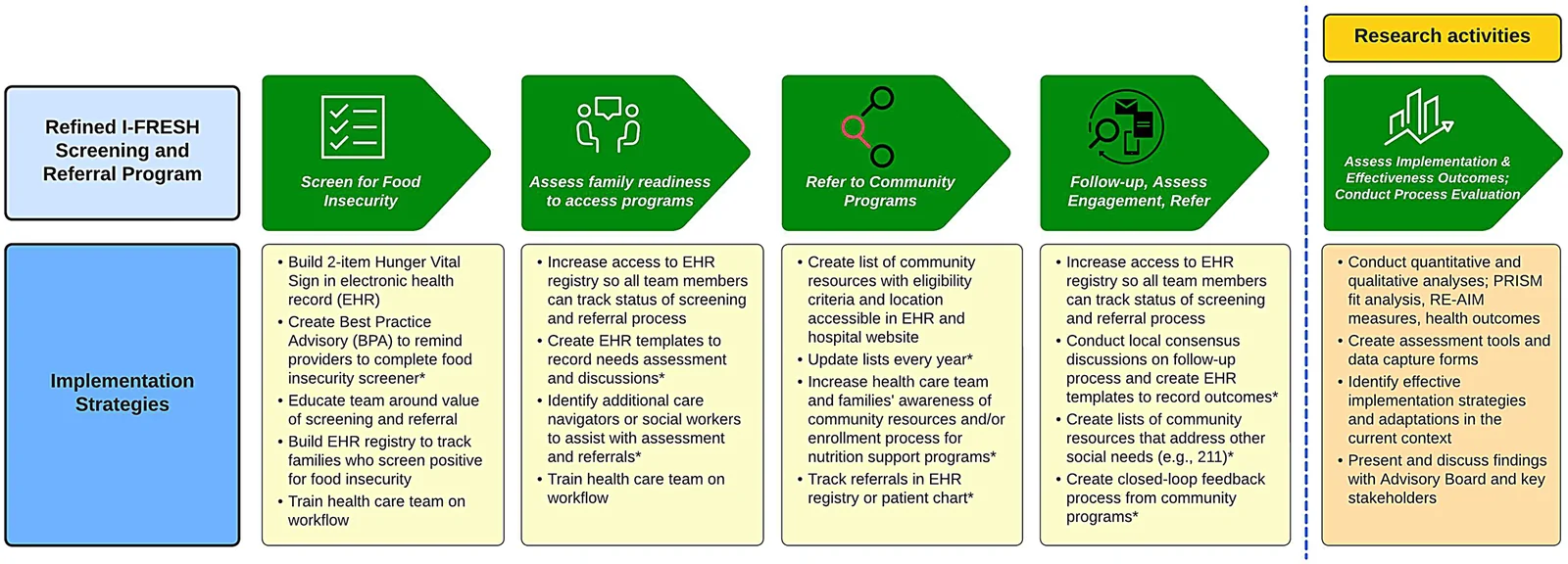
I-FRESH intervention components and implementation strategies. The planned intervention components are listed across the top in green. Below each intervention component are the planned implementation strategies. Proposed implementation strategies that may need further refinement are indicated with a “*”. All components and strategies may be modified during the refinement and optimization process. The Pragmatic, Robust Implementation and Sustainability Model (PRISM) and Reach, Effectiveness, Adoption, Implementation, and Maintenance (RE-AIM) framework will be used to guide the implementation process and evaluation

The purpose of the study is to refine, adapt, and optimize a screening and referral process to address FI in the pediatric healthcare setting. The proposed **I-FRESH (Implementing Food Referrals for Equity and Sustained Health)** program involves screening for FI and referring families to appropriate nutrition support programs in the community. Given that the I-FRESH workflow has not been fully developed or tested in its entirety, we will refine, adapt, and optimize the full workflow and identify implementation strategies using an iterative process defined by the **Roll-Out Implementation and Optimization (ROIO)** study design (Fig. 1). This study design allows us to identify implementation strategies that work in one clinic, and then refine, adapt, and optimize these implementation strategies in the next iteration of the program in the next clinic. We will optimize this workflow over 4 different clinics within the healthcare system and examine implementation and clinical effectiveness outcomes during each phase of the roll-out. The planned components of I-FRESH include: 1) screening and identification of families experiencing FI in the health care setting; 2) social worker or care navigator-led discussions with families to determine need and readiness to receive support; 3) referrals and assistance from allied health professionals or care navigators to engage with community programs and resources; and 4) follow-up assessments to determine program fit, track utilization, and determine need for additional referrals. The implementation strategies will: 1) use tools available in the electronic health record (EHR) to increase efficiency and scalability, and 2) address communication and care navigation failures (Fig. 2).

## PRISM/RE-AIM implementation framework

We will use the **Pragmatic, Robust Implementation and Sustainability Model** (**PRISM**) to guide refinement, optimization, implementation, and evaluation [28, 29]. PRISM allows for systematic, multi-level consideration of factors and contexts that are relevant for implementation success, such as characteristics of program recipients, intervention characteristics, implementation and sustainability infrastructure within the healthcare system and nutrition support programs, and the external federal and state environment that create guidelines and regulations. Refinements and adaptations can then be made to increase the likelihood of program sustainability. By considering the inner and outer contexts that influence how and in what form I-FRESH will be implemented [30, 31], we will outline an effective implementation process model for our healthcare system that addresses FI and provides added health benefits for low-income patients with nutrition-related conditions.

The **Reach, Effectiveness, Adoption, Implementation, and Maintenance** (**RE-AIM**) outcomes framework [32, 33] will be used to evaluate core implementation outcomes, including feasibility, acceptability, fidelity, adoption, reach, and effectiveness. More specifically, we will evaluate the extent to which the intervention reaches eligible patients, is adopted or accepted by different clinical staff, can be delivered with fidelity after adaptations, and can be integrated and sustained in routine pediatric healthcare. Clinical effectiveness outcomes include impact on family-level FI and child health-related outcomes. Together, PRISM and RE-AIM enable a comprehensive, real-world assessment of implementation determinants, processes, and clinical effectiveness.

The specific aims of this study are:

1. To engage our Community, Clinical, Administrative, and Research Advisory Board (CARAB) to *refine and optimize* the program before and after each roll-out in 4 clinics and identify factors in the inner and outer context that serve as barriers and facilitators to engaging in the program.
2. To assess *implementation outcomes* of our comprehensive nutrition support program and determine:
  - *Feasibility and acceptability* of I-FRESH by families, healthcare providers, ancillary support staff, administrators, community nutrition support programs, and community members, and
  - *Fidelity, adoption, and reach* of the program by assessing which components were carried out by ancillary staff/care navigator, staff/setting characteristics, and percentage of families who participated.
3. To examine *preliminary effectiveness outcomes* on family-level FI and pediatric nutrition-related health outcomes, such as, weight status, blood pressure, lipids, HbA1c, and liver function tests. We will also assess changes in medication dose or type (e.g., stopping insulin and only requiring metformin) and engagement in recommended services (e.g., decrease in no-show rates).

We hypothesize feedback and adaptations will develop an implementation bundle that is feasible and acceptable to families, staff, healthcare providers, hospital administrators, and community nutrition support programs. Our goal is to have a robust, feasible, and scalable program that can be tested in a fully powered Type 2 Hybrid Effectiveness-Implementation Trial.

## Methods

Using the ROIO design, we will engage our CARAB to refine and optimize implementation of the I-FRESH program before and after each roll-out in 4 clinics at Rady Children’s Health in San Diego: diabetes, gastroenterology, preventive cardiology, and severe asthma clinic. Periodic Reflections [34] will be held before, during, and after implementation in each of the 4 clinics with CARAB to determine which refinements or adaptations should be made to increase adoption and improve implementation of this workflow. Using the PRISM framework, this group will identify factors in the inner and outer context that serve as barriers and facilitators to I-FRESH implementation and family engagement in the program (Aim 1). Using Implementation Mapping [35] processes, we will refine an effective implementation process model for our healthcare system. We will also assess implementation outcomes of the I-FRESH program using the RE-AIM outcomes framework [32, 33] (Aim 2). Finally, we will examine preliminary effect on family-level food security and pediatric nutrition-related health outcomes (Aim 3). The I-FRESH study was approved by the UC San Diego IRB (protocol # 810869).

### Setting and context

Participants will be drawn from the 4 clinics at Rady Children’s Health in San Diego, a comprehensive pediatric healthcare system serving more than 91% of the children and adolescents (>900,000 youth) in San Diego County (5th largest county in the U.S.), Imperial, and Riverside Counties. In 2022, 22.7% of residents living in San Diego County were below 200% of the Federal Poverty Level (FPL) [36]. Community health assessments conducted by the public health departments of San Diego and Riverside counties identified that low-income households have insufficient resources to buy adequate fruits and vegetables (FV) [37, 38], and 37% to 45% of low-income families with children (<200% of the FPL) have FI [38, 39]. Rady Children’s Health uses Epic^©^ as their electronic health record (EHR), and the 2-item Hunger Vital Sign [40] was implemented in 2020. The health system has a SDOH workgroup that consists of physician champions, Information Technology (IT) experts, dietitians, social workers, and members of the Population Health department. This workgroup assists in making changes in Epic^©^, creating decision support tools, and addressing other hospital-wide procedures and regulations that hinder or assist efforts to identify and address SDOH needs of the patients.

### Participants

Members of the CARAB (which includes health care providers, hospital administrators, population health/public health leaders, staff from community-based organizations (CBOs), food as medicine experts, equitable food systems collaborative leaders, community members, community health workers, and the research team) will be invited to provide their views and opinions on how the program is functioning and complete RE-AIM assessments. Our goal is to recruit up to 100 health care staff and CARAB stakeholders to complete Aims 1 and 2. Up to 10 parent participants per clinic (*n* = 40) who are 18 years or older will also be asked to participate in Aims 1 and 2. These parents/caregivers will have a child with a nutrition-related condition who attends one of the 4 participating clinics and screens positive for FI.

To study the impact of this program on family-level FI and pediatric nutrition-related health outcomes (Aim 3), we plan to recruit 240 parents of a child aged 0–18 attending one of the participating clinics (~60 per clinic). These families will screen positive for FI based on the 2-item Hunger Vital Sign [40].

Exclusion criteria will be limited given the heterogeneous nature of the populations in each clinic. This will increase generalizability of our findings and allow us to explore a wide range of implementation issues before testing this program in a larger type 2 hybrid effectiveness-implementation trial. We will track the following variables and include them as covariates or moderators rather than exclusion criteria: 1) child medical conditions for which physician supervision of diet are needed; 2) child body mass index (BMI) percentile; 3) child medications; 4) family language preferences; 5) family household size and composition; and 6) other SDOH factors (e.g., lack of housing or transportation) that would impact the family’s ability to access nutrition support programs.

### Study objectives

#### Specific Aim 1: Refinements and adaptations: implementation Mapping

The goal for Aim 1 is to identify which components of the I-FRESH intervention and implementation strategies are effective and which intervention components and implementation strategies need to be modified to increase the impact of I-FRESH for different populations in different clinic settings. This information will help to identify which program components and implementation strategies are key to program success and should be prioritized when implemented across diverse settings and external contexts. We will use the Implementation Mapping [35] approach to develop expected implementation outcomes for the RE-AIM domains of adoption, implementation, fidelity, and maintenance.

Prior to the first roll-out, we will engage CARAB and other key stakeholders to participate in a brainwriting premortem [41] discussion to refine the program structure and identify potential barriers and facilitators to implementing the full workflow. During this this co-production process [42], participants with expertise in implementation science, clinical informatics, behavior change, and historical evidence behind implementing such a program will be able to discuss with those who have detailed knowledge of the logistics of implementing such a program in the hospital and its EHR, and those who have expertise around the community/family perspectives and context. During this process, modifications to implementation strategies, program content, format of delivery, and timing of program components will be made as needed. Barriers to optimizing fidelity and reach (be it from the internal or external context/environment) will be identified, documented, and addressed when possible. Program materials and protocols will be adapted to foster multiple levels of communication and information sharing between families, the health system, and community programs. Finally, existing materials, referrals, and resources will be modified to make them more user friendly for the English and Spanish-speaking populations. Members of the CARAB will provide input on all these modifications and review program materials before they are implemented.

Once the program is implemented in each clinic, we will conduct periodic reflections [34] with key stakeholders, including families, and the CARAB to allow for real-time iterations in program implementation. These reflections will occur before, during, and after roll-out of the intervention in each of the 4 clinic sites. Roll-outs in each new clinic will occur every 4 months. We will use a modified Stirman Framework [43] to track adaptations monthly [44]. Documentation of adaptations will include brief multilevel surveys with staff and key stakeholders. Semi-structured interviews or focus groups with key stakeholders and families will occur at 3 and 6 months to expand on written adaptations. Data from all sources will be triangulated using joint display analysis [45] to determine which adaptations should be included as permanent implementation strategies in the next clinic iteration

**Proposed I-FRESH workflow and implementation strategies:**

The I-FRESH workflow includes: 1) Screening and Identification; 2) Readiness assessment, 3) Referrals; 4) Follow-up regarding access, utilization, and fit of referral service. (Fig. 2)

##### Screening and identification

The first step in this process is to identify FI through regular surveillance and screening with validated screening tools [46]. We plan to use the EHR to implement the screening tool because it has been shown to improve SDOH reporting compared to in-person assessments that may occur in the presence of children or other family members, and lead to shame or embarrassment [47–49]. However, this strategy may need to be refined to include reminders or prompts if the screener has not been completed. This process also assumes that families are interested in receiving help in this domain [25, 50]. Some families may have other priorities that need to be addressed first [26, 51]. Other families may not report FI status because of concerns that information is being shared with law enforcement or government agencies [26, 51]. Families may also fear discrimination from healthcare providers for being identified as having FI and carry their own personal shame around these issues [52]. For these reasons, we will engage with CARAB and other key stakeholders in the healthcare system to develop a process where family/patient needs and interests are reviewed in a manner that allows for personal autonomy and is responsive to their current situation. Before each roll-out, we will educate the healthcare team on this newly refined process. An EHR registry will also be created to track families who have screened positive for food insecurity to facilitate next steps in the screening and referral process.

##### Readiness assessment

In-person assistance with referrals and resource navigation has been shown to result in higher contact or follow-up with identified services [53]. Therefore, discussions with the health care provider (physician, nurse practitioner, nurse) or social worker, care navigator, or community health worker will be included to allow for assessment around urgency of need and readiness to utilize community support programs. It also provides an opportunity to address concerns or questions about the referrals. The EHR registry will be used to track which families need additional follow-up or care navigation. However, refinements to this implementation strategy may be needed to determine which members of the healthcare team will conduct this follow-up and track the family’s progress. Ultimately, linking these conversations (which would include a shared decision-making model and trauma-informed lens [24, 54]), with the screening process may facilitate enrollment into nutrition support programs [55]. These conversations will also allow the health care provider and/or allied health professional to assess the patient or family’s assets and strengths [56–58], and highlight them as tools to mitigate risk.

##### Referrals

In addition to developing a robust screening and identification process, it is important to identify relevant resources to address discovered SDOH needs. Screening without offering assistance may cause distress for both affected families and health care providers, resulting in a failed program. Some families may not reveal they have SDOH needs unless resources are provided [50, 59], or there is some indication that services are available if needed. Thus, in our program, we plan to inform families there are resources available for those who are interested before they complete the FI screener. We plan to develop a repository of community resources that is available to staff and families in the EHR and on the health system website. Refinements will need to be made to determine the best strategies to increase staff and family awareness of these resources.

##### Follow-up: access, utilization, and fit of referral service

After the initial referral, care coordinators will follow-up with families within 2–4 weeks to obtain feedback regarding the programs or CBOs they accessed, and problem-solve any challenges. This workflow is based on prior best practice we developed with the San Diego County 211 program. However, we will need to determine which strategies to utilize to implement this component of the intervention. Local consensus discussions may need to occur to determine how feasible this is. During this follow-up call, it is proposed that care coordinators will determine whether families contacted the program and if they encountered any barriers. They will also collect feedback about the CBO program and identify if there are other needs. Additional referrals will be made if needed. Depending on the feedback received during the implementation and adaptation process, this component may be repeated every 2–4 weeks until families no longer require the help of the care coordinator.

#### Specific Aim 2: Assess implementation outcomes

With each iteration of the roll-out, we will gather implementation outcomes from each level of the program: individual/family, hospital, and community partner level. Using the RE-AIM outcomes framework [32, 33], our primary implementation outcomes are feasibility and acceptability of I-FRESH by families, healthcare providers, ancillary support staff, administrators, community nutrition support programs, and community members. We will also assess fidelity, adoption, and reach of the program by assessing which components were carried out by ancillary health care staff/care navigator, staff/setting characteristics, and percentage of families who participated. Reach and maintenance of the program will be tracked through the EHR registry. (Further details on assessments are presented below in Measures.)

#### Specific Aim 3: Examine preliminary impact and effectiveness outcomes

We will further apply RE-AIM and examine exploratory clinical effectiveness outcomes including family-level FI and pediatric nutrition and metabolism-related health outcomes that may be similar across the different clinics: weight status, blood pressure (BP), lipids, HbA1c, and liver function tests. We will also assess changes in medication dose or type (e.g., stopping insulin and only requiring metformin) and engagement with the clinic or recommended services (e.g., decrease in no-show rates). While the larger goal of the program is to decrease FI and improve health outcomes, we will also collect information on the family’s self-reported knowledge, skills, attitudes (expectancies), self-efficacy, and social support needed to engage in the program. Other behaviors of interest include consumption of and barriers to consuming fruits and vegetables.

### Measures: (Table 1)

#### Implementation adaptations and outcomes (Aims 1 and 2)

We will use several data collection methods to capture the adaptations made to the implementation strategies (Aim 1), such as the modified Stirman adaptation framework and coding system [44, 69] to track refinements that occur. We will ask clinic staff and other hospital or program-related staff to record any adaptations that have been or need to be made in program content or delivery. Then we will review this in CARAB meetings where periodic reflections [34] will occur to determine if iterative refinements need to be made to the program before the next roll-out. These reflections will code the who, how, what, when, and why the adaptation was made, and barriers or facilitators (program determinants) to program implementation. These reflections should address the following questions (among others): What are the contextual contingencies that would make this program implementation more successful, what resources (e.g., personnel, IT support) are needed to successfully implement this program, what adaptations to the workflow need to be made, how willing is the current workforce to make these adaptations, how can we fit the program workflow into current contextual workflows? Additionally, semi-structured interviews will be conducted with multiple family, community, clinical, and hospital level stakeholders to determine if other refinements should occur and the perceived importance of these and other previously identified modifications. We will use the FRAME-IS [70, 71] (which assesses what types of adaptations were made and why) as prompts for these interviews/meetings and review any immediate changes needed at the research team and CARAB meetings. Solutions to the identified barriers will be co-created during these meetings.

**Table 1:** Assessments for each Aim and level (family, healthcare system, community program)

	Assessments	Parent (P), Child (Ch), Healthcare (H), Community (Com)	Data type & source	Frequency of assessment
Aim 1 Implementation Mapping and Optimization	• Adaptations and Implementation strategies • Barriers to program implementation • Structures and systems needed to implement program and support community programs • Experience with multi-component implementation • PRISM Fit assessment	H, Com H, Com H, Com P, H, Com P, H, Com	Periodic reflection, Interviews Periodic reflection, Interviews	Before, during, after each roll-out Before, during, after each roll-out
Aim 2 Implementation Outcomes	• Acceptability, Feasibility, Appropriateness of program [60] • Reach: % of families attending clinic that completed screener, # of families in the registry, % of families that did not want service, Characteristics of participating vs. non-participating families • Adoption: Characteristics of clinic settings and staff, Characteristics of community program (location, months/days/hours of operation, description of benefits or incentives, educational programs available) • Readiness for organizational change scale [61] • Implementation Climate Scale [62] • Implementation/Fidelity: % of families that were called, % of families that were referred or provided with resources, % of families that received follow-up phone calls, % of families that needed additional resources/referrals • Maintenance: • Program Sustainability Index [63] • Perceived support of innovation in organizations [64] • Partnership synergy tool [65–67]	P, H, Com H H, Com H H, Com	Periodic reflection, Interview, Survey EHR, Survey Survey, Periodic reflection EHR, Periodic reflection Survey	6, 12 mos Ongoing & 12 mos Ongoing & 12 mos Ongoing & 12 mos 12 mos
Aim 3 Effectiveness Outcomes	• Health outcomes: BMI/weight status, HbA1c, blood pressure, total cholesterol, HDL cholesterol, liver function, health status • Quality of life, stress, well-being • PRAPARE (patient assets, risks, and experiences) • Medications (including dose) & Health care utilization • Community program utilization (including barriers & facilitators) • Knowledge, Skills, Attitudes, Self-efficacy • Food Frequency Questionnaire [68] • Demographics: Race/ethnicity, age of child and parent, sex and gender identity of child and parent, address, zip code, birthplace, current employment status, educational attainment, annual family income, health insurance • SDOHfactors: Food insecurity, health literacy & numeracy, access to health services, access to health technology, internet access, job insecurity, housing insecurity, discrimination, discrimination in health care, food swamp	Ch P P Ch P P P P, Ch P	EHR Survey Survey EHR Survey Survey Survey Survey, EHR Survey	0, 6, 12 mos 0,12 mos 0,12 mos 0,12 mos 0,12 mos 0, 6, 12 mos 0, 6, 12 mos 0 mos 0, 12 mos

The PRISM Fit Assessment [72–74] will be conducted before each new roll-out of a clinic which examines how well the current implementation strategies align with the new clinic and whether it will reach similar RE-AIM metrics as in the previous clinic implementation. Representative clinic providers and staff, community stakeholders and hospital administrators will complete this 21-item survey using the iPRISM web tool (prismtool.org) before each clinic roll-out. Results will be reviewed during a CARAB meeting and additional refinements to the implementation strategy will be made.

Using the RE-AIM framework [32, 33], we have identified several implementation outcomes to assess (Aim 2). These variables include reach, adoption, maintenance, acceptability, appropriateness, and feasibility of implementation strategies. We will be able to track reach and maintenance of the program through the registry we create in the EHR. All patients attending the participating clinics will be included in the registry, and we will be able to track what proportion completed a FI screener, and what proportion screened positive for food insecurity. This data can be visualized in a dashboard and used to provide feedback to key stakeholders until it is disabled. To better understand factors from the external and internal context that support implementation and adoption, we will assess the readiness of the health care setting, community-based organizations (CBOs), and individuals participating in this program. Finally, characteristics of the health care setting, community programs, and families that increase and decrease the likelihood that adopting this food security referral program model will be assessed.

#### Program acceptability, feasibility, and process evaluation

Quantitative and qualitative assessments of intervention acceptability and satisfaction will be collected from parents and providers/staff. For parents, we will administer self-report surveys (*n* = 60 per clinic) after they receive referrals and engage in nutrition support programs for 6- and 12-months. A random sample of parents (*n* = 10 per clinic, using a purposeful sampling method to include parents who successfully accessed programs and those who did not) will be invited to participate in a post-interview. The post-interviews will be conducted using a semi-structured interview guide probing relevant PRISM domains such as parents’ experience, usefulness, satisfaction, and recommendations for improvements with each program component, including concerns or barriers to accessing the CBOs, and interactions with health care providers, care navigators, and CBOs.

Providers, care navigators, and key health care administrators will also be invited to participate in a 45–60 minute group-based periodic reflection [34] (per active clinic) to determine challenges, opportunities, and modifications needed to optimize program implementation for the subsequent clinic roll-out. All participants will be invited to complete post-roll-out surveys (every 6 months) to assess impact, feasibility, acceptability, and appropriateness or fit of the program using the 4-item Acceptability of Intervention Measure (AIM), Intervention Appropriateness Measure (IAM), and Feasibility of Intervention Measure (FIM)) [60]. All interviews and periodic reflections will be audiotaped and transcribed verbatim for analysis.

At the community partner level, focus groups or key informant interviews will be conducted to determine the impact of referrals on their program (e.g., were they able to meet the increased demand) and what further improvements in communication are needed between the health care system and families. Final workflows and systems will be reviewed and refined by community stakeholders before being utilized in a fully-powered clinical trial.

#### Program fidelity and reach

Fidelity to intervention components and implementation outcomes will be tracked. Care navigators will document the following: whether they were able to contact a family who screened positive for FI, whether the parent was open to receiving referrals to nutrition support programs, whether follow-up phone calls were made to check on progress and program utilization, and whether additional referrals were needed. This information will also help us to determine the reach of the program and how many families were able to participate compared to the overall number of families who screened positive for FI.

#### Medical record abstraction

EHR data will be used to track preliminary clinical effectiveness outcomes (Aim 3). EHR data will be used to track patient health outcomes (e.g., BMI, HbA1c, LFTs, cholesterol), SDOH outcomes, medication adherence, health care utilization (e.g., follow-up visits made to primary care or subspecialty physicians, adherence to appointments, ED visits, hospitalizations, lab orders, medications prescribed), and I-FRESH program process measures (e.g., phone call attempts by the care navigator, referrals made to community nutrition support programs). Demographic information (i.e., age, sex, race, ethnicity, insurance status) will be collected to examine if there are differences between those who engage in the program and those who do not.

### Statistical analyses

#### Qualitative data and mixed methods integration

Qualitative data from the family interviews and periodic reflections will be analyzed using a rapid qualitative data analytic approach guided by the domains of PRISM [29]. Specifically, we will develop a matrix of summary responses from the qualitative data sources. A codebook will be developed from this matrix and used on all transcripts, allowing for both a priori and emergent themes. Coding will be completed by a qualitative analytic team and a small group of trained research team members. A subset of data will be group coded for consensus-building followed by independent coding, team-based review of codes, and further refinement of the codebook, as needed. The rest of the transcripts will be independently coded with 10% of them being double coded to verify acceptable inter-rater reliability (>70% consensus). We will also engage the CARAB group in reviewing the coded data to support sense-making and develop action items based on the data to identify refinements prior to each rollout. We will use a joint display analysis [45] to support the integration of survey and qualitative data sources for the purposes of comparison, complementarity, and expansion [75–77].

#### Adaptations documentation data

We will use the Stirman et al. [43, 70, 71] Adaptations framework (FRAME-IS) to characterize adaptations identified throughout the study period using the adaptations tracker. The research team will meet monthly to review the Adaptations tracker and determine whether any adaptation requires immediate action to support implementation optimization.

#### Quantitative data

Primary analyses for Aim 2 will assess fidelity and reach outcomes (Aim 2b) assessed within the EHR (e.g., screening, referrals, and follow-up assessments) and surveys (e.g., Acceptability, Feasibility, and Appropriateness) across the study period using Generalized Linear Mixed-effects Models (GLMM) and time-varying indices for clinics randomized to cross-over in each wave, time (secular trend), and clinic characteristics as planned covariates. Serial cross-sectional summaries for each clinic within monthly blocks will allow estimation of changes in clinic outcomes after crossing over into interventions. Primary analyses for Aim 3 will describe I-FRESH participants’ (*n* = 240) changes in health outcomes including: a) metabolic labs, BP, BMI, b) healthcare utilization; c) community program utilization; d) Quality of Life; e) Food Frequency Questionnaire and f) Knowledge, Skills, Attitudes, and Self-Efficacy. GLMM growth models for individual trajectories of continuous and limited categorical outcomes will include a focus on significance of changes from baseline to post-treatment assessments after graphically examining an appropriate functional form (e.g., linear or categorical) for modeling each outcome. All models will include participants’ characteristics (gender, ethnicity, parental education) and allocation clinic as planned covariates when estimating changes in primary health and engagement outcomes. We will explore the role of SDOH and implementation strategy effectiveness as potential influences in describing changes in parent and child health outcomes. Benjamini-Hochberg [78] procedures will be used to adjust *p*-values for inference decisions within common domains.

##### Missing data

Patient loss to follow-up is not uncommon in the primary care setting. Mitigation efforts include appointment reminders, follow-up by care navigators, and options for telemedicine visits. A 20% loss to follow-up has been factored into our sample size estimation. For covariates or confounders which are missing at 10% prevalence or less we will use multiple imputation (mice) [79] or related EM likelihood-based procedures for evaluations in Aim 3 [80].

##### Power considerations

We conducted power/sample size for this ROIO design trial to address primary aims, using developed formulas [81] drawn from stepped wedge designs that account for unequal cluster size and package *swCRTdesign* [82]. The design includes 4 identified clinics (clusters), at least one 3-month period in standard care and 4 additional periods where assigned clinics can transition to I-FRESH at 4-month intervals. We estimated the needed sample to test a 10% difference in proportions of engagement in services (i.e., Aim 2b healthcare and community program utilization), and a standardized mean increase of at least d ≥ 0.20 when reported at 6- and 12-month health outcomes. With alpha = 0.05, 4 clusters (1 clinic per cluster = 4 clinics with *n* = 300 - 500 potential patients in each clinic) and five periods of transitions (baseline and 4 clinics), we will maintain power > 0.83 to detect clinic differences (I-FRESH vs standard care) of >8% in rates of screening, referral and follow-up engagement in services and changes in acceptability ratings of d > 0.20 after implementing I-FRESH given a between clinic standard deviation of 0.05 in random treatment effects. Using an empirical approach, we simulated 1000 data sets with expected mean changes (d ≥ 0.20) and serial correlations similar to pilot samples. We used the percentage of growth models with *p* < 0.05 for slope parameters to estimate power given proposed sample of 60 per clinic and allowance for 20% missing data. With median slope of 0.02 (increase per month; estimate sd = 0.01) we observed significant effects in >81% of models suggesting sufficient power to detect desired effects in Aim 3 with the proposed sample.

### Dissemination plan

Data will be shared in ClinicalTrials.gov. We will also share information with key Rady Children’s Health leaders and staff to ensure adoption and maintenance of the I-FRESH implementation bundle. Results will also be shared with CBOs and other community stakeholders to sustain connections between the healthcare system and community, and foster continued development of robust collaboration models. Results from this trial will be submitted to peer-reviewed scientific journals and relevant national conferences. All publications will be available on PubMed and the NIH Manuscript Submission system.

## Discussion

While identification of adverse SDOH conditions is an increasingly important activity among healthcare providers and systems, workflows that include adequate screening and referrals to programs can be complex, difficult to implement, and nuanced based on the available institutional infrastructure, culture, and environmental/community context. The goal of this study is to help identify effective workflows and implementation strategies that can increase adoption and maintenance of these activities.

Access to good nutrition has been shown to have positive impacts on physical and mental health outcomes. However, systematic approaches to facilitating access to nutrition support programs have not always been fully integrated within healthcare systems. Nevertheless, healthcare systems are starting to adopt universal SDOH screening and create programs such as fruit and vegetable prescription (FVRx) programs (funded by state, federal, or philanthropic organizations) to support the identified needs [83].

The development of multi-level programs that include multi-sector community collaborations are essential to address FI [84, 85]. These activities include government, community, and private business involvement across multiple sectors (e.g., health care, housing, transportation, food access, environment) [86]. The implementation model developed in this project will help to identify key levers that should be addressed in order to create a sustainable model for healthcare systems and communities. This information will be critical as health system leaders, insurance providers, community programs, public health agencies, and policy makers strive to integrate these programs into our healthcare system.

## Data Availability

No datasets were generated or analysed during the current study.

## Abbreviations

FI: Food Insecurity
T2D: Type 2 diabetes
CVD: Cardiovascular disease
SNAP: Supplemental Nutrition Assistance Program
SDOH: Social determinants of health
I-FRESH: Implementing Food Referrals for Equity and Sustained Health
FV: Fruits and vegetables
ROIO: Roll-Out Implementation Optimization design
PRISM: PragmaticRobust Implementation and Sustainability Model
RE-AIM: Reach, Effectiveness, Adoption, Implementation, and Maintenance
CARAB: Community, Clinical, Administrative, and Research Advisory Board
EHR: Electronic health records
CBOs: Community-based organizations
HbA1c: Hemoglobin A1c
FVRx: Fruit and vegetable prescription program
